# TGF-β1/SMOC2/AKT and ERK axis regulates proliferation, migration, and fibroblast to myofibroblast transformation in lung fibroblast, contributing with the asthma progression

**DOI:** 10.1186/s41065-021-00213-w

**Published:** 2021-12-08

**Authors:** Yuebin Wang, Huike Yang, Xian Su, Anqiang Cao, Feng Chen, Peng Chen, Fangtao Yan, Huirong Hu

**Affiliations:** 1 Department of Respiratory and Critical Care Medicine, Chengdu Third People’s Hospital, Chengdu City, 610031 Sichuan Province China; 2 Department of Anatomy, Harbin Medical University, Harbin City, 150081 Heilongjiang Province China; 3 Department of Cardiothoracic Surgery, Chengdu Third People’s Hospital, No.82, Qinglong Street, Qingyang District, Chengdu City, Sichuan Province China

**Keywords:** SMOC2, TGF-β1, Myofibroblast transformation, Lung fibroblasts, Asthma

## Abstract

**Background:**

Asthma is a common chronic respiratory disease that influences 300 million people all over the world. However, the pathogenesis of asthma has not been fully elucidated. It has been reported that transforming growth factor-β (TGF-β) can activate myofibroblasts. Moreover, the fibroblast to myofibroblast transformation (FMT) can be triggered by TGF-β, which is a major mediator of subepithelial fibrosis. Secreted modular calcium-binding protein 2 (SMOC2) is a member of cysteine (SPARC) family and is involved in the progression of multiple diseases. However, its role in asthma remains poorly understood. RT-qPCR evaluated the expression of SMOC2. Bromodeoxyuridine assay and wound-healing assay detected the proliferation and migration of lung fibroblasts, respectively. IF staining was performed to assess the expression of α-smooth muscle actin (α-SMA). Western blot analysis detected the levels of proteins. Flow cytometry was utilized for determination of the number of myofibroblasts.

**Results:**

We found the expression of SMOC2 was upregulated by the treatment of TGF-β1 in lung fibroblasts. In addition, SMOC2 promoted the proliferation and migration of lung fibroblasts. More importantly, SMOC2 accelerated FMT of lung fibroblasts. Furthermore, SMOC2 was verified to control the activation of AKT and ERK. Rescue assays showed that the inhibition of AKT and ERK pathway reversed the promoting effect of SMOC2 overexpression on proliferation, migration and FMT in lung fibroblasts.

**Conclusions:**

This work demonstrated that SMOC2 modulated TGF-β1-induced proliferation, migration and FMT in lung fibroblasts and may promote asthma, which potentially provided a novel therapeutic target for the management of asthma.

**Supplementary Information:**

The online version contains supplementary material available at 10.1186/s41065-021-00213-w.

## Background

As a common chronic respiratory disease, asthma seriously influences nearly 300 million people all over the world [1]. Reversible airway obstruction, airway inflammation and hyperresponsiveness are features of asthma in clinic. Asthma is common among children and young people globally, which has a heavy burden on health systems and causes a loss of productivity [2]. However, the pathogenesis of asthma has not been fully elucidated up to now.

The basilar membrane in the bronchial mucosa of asthmatic patients may be thickened to different degrees [3–6], which may result from the deposition of collagen and adhesion proteins and further leads to the proliferation of myofibroblasts [7]. Myofibroblasts are cells that can express α-smooth muscle actin (α-SMA) and secret extracellular matrix (ECM), which are vital for tissue contraction and remodeling [8]. Myofibroblasts mainly originates from resident fibroblasts [9]. A previous study has showed that transforming growth factor-β (TGF-β) can activate the myofibroblasts and increase myofibroblast proliferation [10]. Moreover, the fibroblast to myofibroblast transformation (FMT) can be triggered by TGF-β, which is a major mediator of subepithelial fibrosis and upregulated in asthmatic bronchi tissues [11, 12]. Therefore, it is of huge importance in the pathogenesis of asthma.

Secreted modular calcium-binding protein 2 (SMOC2) is a member of secreted protein acidic and rich in cysteine (SPARC) family [13]. SMOC2 is a matricellular protein that has been reported to express in various tissues and participates in different cellular processes, including cell migration [14, 15]. Previously, several studies have revealed that SMOC2 is involved in the progression of multiple diseases. For instance, SMOC2 downregulation decreases bleomycin-triggered pulmonary fibrosis via the inactivation of TGF-β1/SMADs pathway [16]. SMOC2 aggravates kidney fibrosis through the inhibition of the progression of FMT [17]. Besides, SMOC2 has also been recognized as a prognostic marker of papillary thyroid carcinoma [18]. Although the function of SMOC2 is confirmed in several diseases, its role in asthma remains poorly understood.

In this study, we aimed at investigating whether SMOC2 can participate in TGF-β1-induced proliferation, migration and FMT in lung fibroblasts and further to determine the involvement of SMOC2 in asthma. The results of our study illustrated that SMOC2 was involved in TGF-β1-induced proliferation, migration and myofibroblast transformation in lung fibroblasts, which may contribute to asthma. These findings might offer a novel biomarker for asthma treatment.

## Results

### TGF-β1 induced the upregulation of SMOC2 in lung fibroblasts

**Fig. 1:**
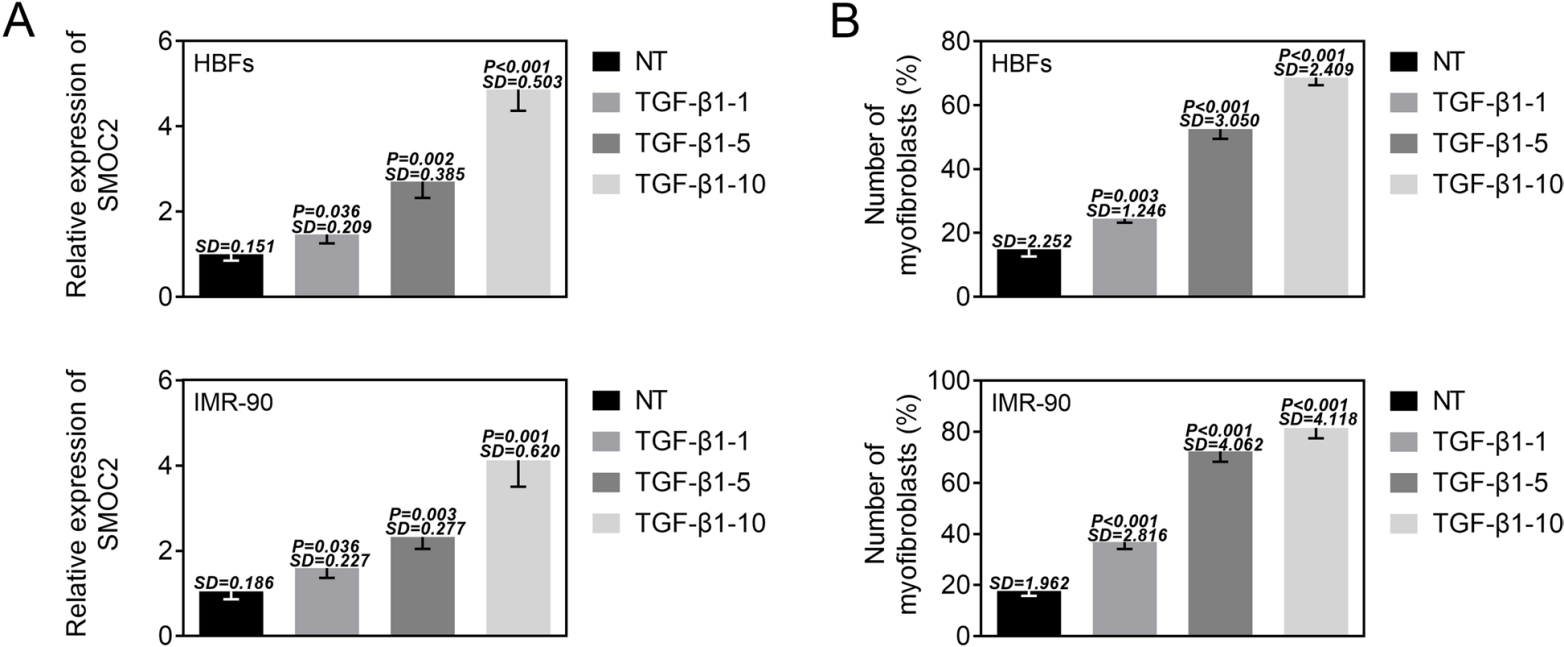
TGF-β1 induced the upregulation of SMOC2 expression in lung fibroblasts. (A) RT-qPCR was implemented to detect the expression of SMOC2 in HBFs and IMR-90 cells following different concentration of TGF-β1 treatment (0, 1, 5, 10 ng/mL). (B) The numbers of myofibroblasts were detected by flow cytometry analysis in HBFs and IMR-90 cells following different concentration of TGF-β1 treatment (0, 1, 5, 10 ng/mL). ^*^p < 0.05 compared with NT group, ^**^p < 0.01 compared with NT group

In order to uncover whether the expression of SMOC2 could be influenced by TGF-β1 treatment in lung fibroblasts, RT-qPCR was implemented to detect the expression of SMOC2. Compared the normal HBFs and IMR90 cells, the expression of SMOC2 in both HBFs and IMR90 cells was enhanced by different concentrations of TGF-β1, especially 10 ng/mL TGF-β1 (Fig. 1A). Meanwhile, the numbers of myofibroblasts in both HBFs and IMR90 cells were elevated by TGF-β1 treatment in a concentration-dependent manner (Fig. 1B). Taken together, TGF-β1 induced the upregulation of SMOC2 in lung fibroblasts and increased the numbers of myofibroblasts, suggesting that SMOC2 was closely associated with the fibroblast to myofibroblast transformation.

### SMOC2 modulated the proliferation and migration of lung fibroblasts

**Fig. 2:**
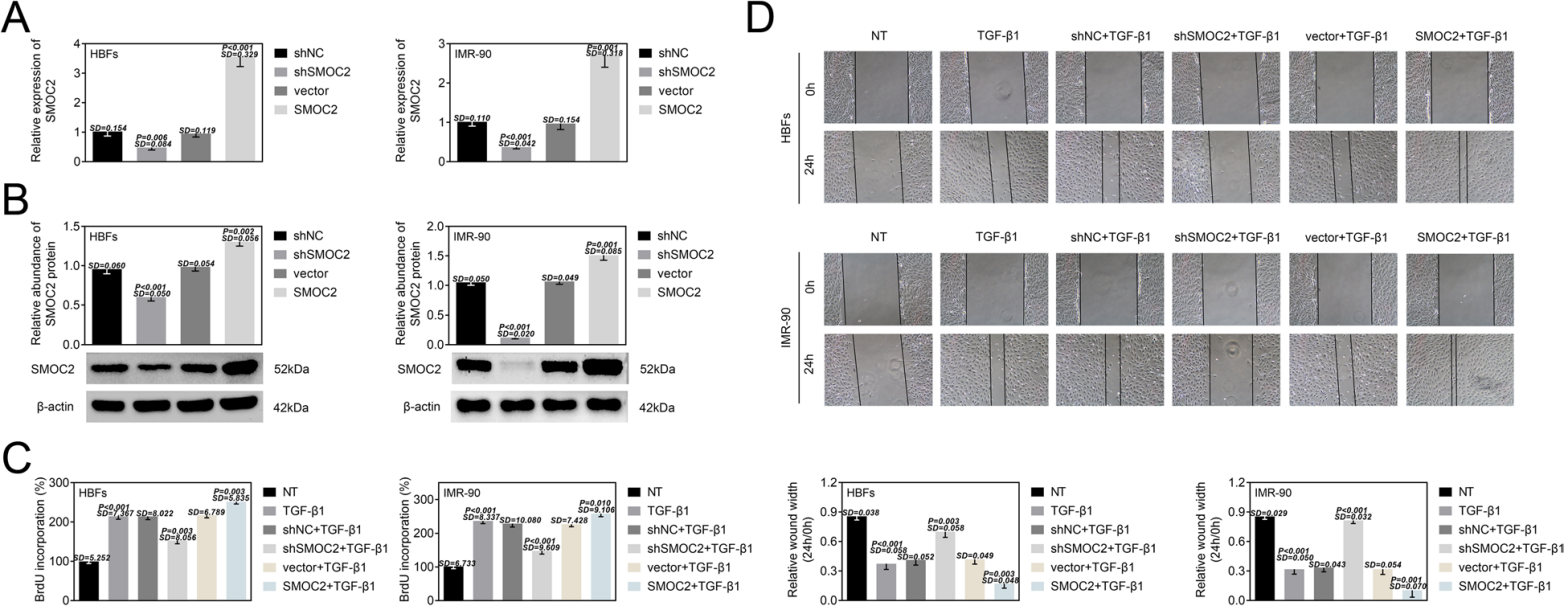
SMOC2 modulated the proliferation and migration of lung fibroblasts. (A) RT-qPCR was carried out to disclose the expression of SMOC2 in HBFs and IMR-90 cells after transfection of shNC, shSMOC2, vector or SMOC2. ^**^p < 0.01 compared with NT group; ^&&^*p* < 0.01 compared with vector group. (B) The protein level of SMOC2 was assessed via western blot analysis in HBFs and IMR-90 cells after transfection of shNC, shSMOC2, vector or SMOC2. ^**^p < 0.01 compared with NT group; ^&&^*p* < 0.01 compared with vector group. (C) BrdU assay was performe to assess the proliferation of HBFs and IMR-90 cells after TGF-β1 treatment or combined with transfection of shNC, shSMOC2, vector or SMOC2. ^**^p < 0.01 compared with NT group; ^&&^*p* < 0.01 compared with shNC+TGF-β1 group; ^@@^*p* < 0.01 compared with vector+TGF-β1 group. (D) The migration ability was detected by wound-healing assay in HBFs and IMR-90 cells after TGF-β1 treatment or combined with transfection of shNC, shSMOC2, vector or SMOC2. ^**^p < 0.01 compared with NT group; ^&&^*p* < 0.01 compared with shNC+TGF-β1 group; ^@@^*p* < 0.01 compared with vector+TGF-β1 group

To further analyze the function of SMOC2 in lung fibroblasts, SMOC2 was firstly silenced or overexpressed in HBFs and IMR-90 cells. The data from RT-qPCR disclosed that the expression of SMOC2 in HBFs and IMR-90 cells was evidently decreased with 0.5- or 0.4-fold change due to the transfection of shSMOC2. SMOC2 overexpression resulted in 3.4- or 2.7-fold increase of SMOC2 expression in HBFs and IMR-90 cells (Fig. 2A). Similarly, the protein level of SMOC2 in HBFs and IMR-90 cells was also downregulated with 0.6- or 0.1-fold change on account of SMOC2 inhibition. The protein level of SMOC2 was 1.3- or 1.4-flod higher in HBFs and IMR-90 cells in the presence of SMOC2 overexpression vector than that in normal HBFs and IMR-90 cells (Fig. 2B). Moreover, we examined the impact of SMOC2 knockdown and overexpression on cell proliferation and migration of HBFs and IMR-90 cells. BrdU assay revealed that TGF-β1 treatment could increase the proliferation ability of HBFs and IMR-90 cells, which was reversed by silencing of SMOC2. However, SMOC2 overexpression further promoted cell proliferation of TGF-β1-treated HBFs and IMR-90 cells (Fig. 2C). In consistent, the migration ability of HBFs and MR-90 cells was increased (wound width: HBFs: from 0.86 to 0.44; MR-90: from 0.84 to 0.27) by the treatment of TGF-β1. TGF-β1-induced promotion of migration ability in HBFs and MR-90 cells was reversed by suppression of SMOC2 (wound width: HBFs: 0.64; MR-90: 0.82). SMOC2 upregulation caused an increase of migration ability (wound width: HBFs: 0.22; MR-90: 0.18) in TGF-β1-treated HBFs and MR-90 cells (Fig. 2D). In summary, SMOC2 promoted the proliferation and migration of TGF-β1-treated lung fibroblasts.

### SMOC2 regulated FMT of lung fibroblasts

**Fig. 3:**
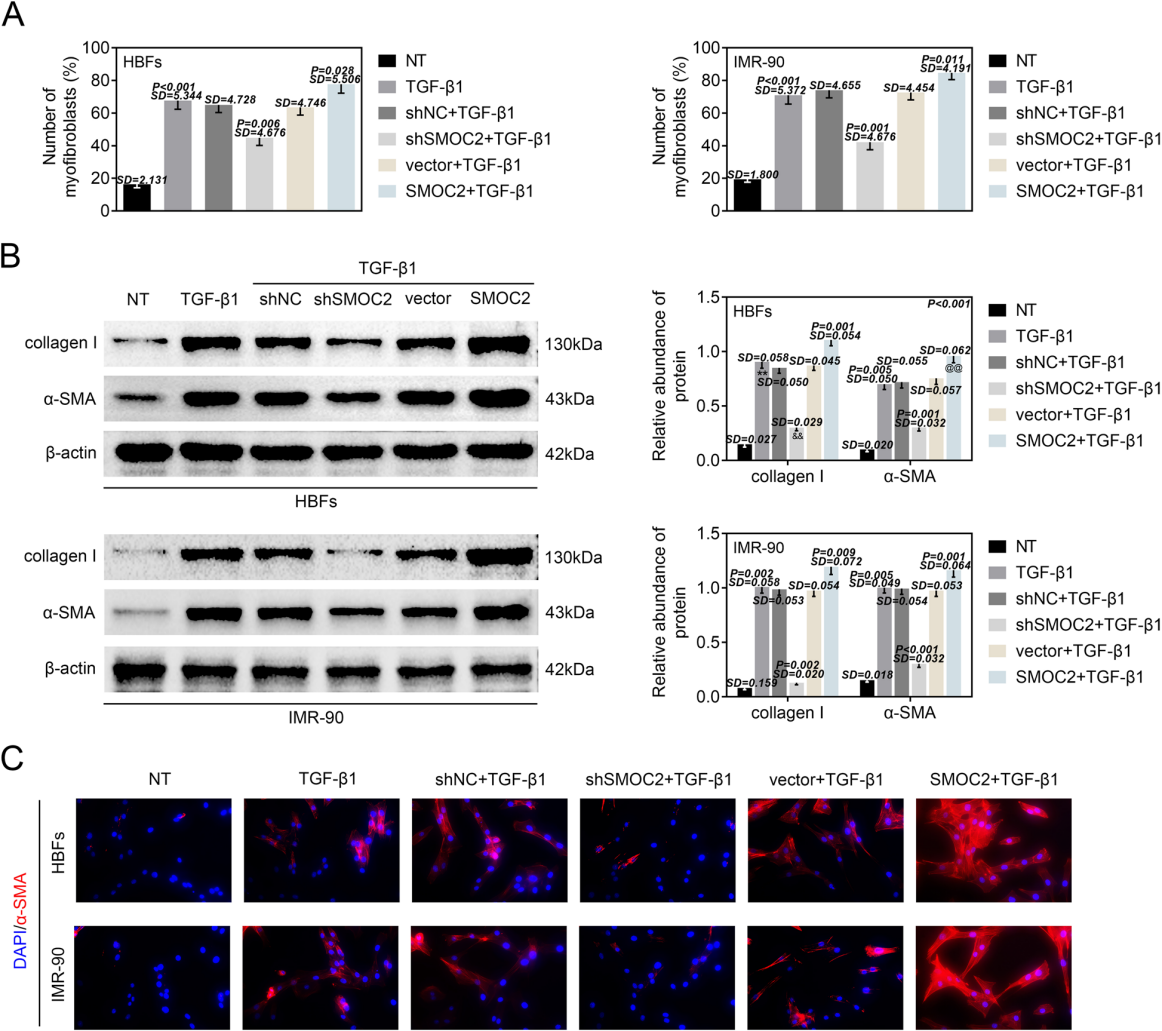
SMOC2 regulated FMT of lung fibroblasts. HBFs and IMR-90 cells were transfected with shNC, shSMOC2, vector or SMOC2, and then treated with TGF-β1. (A) Flow cytometry analysis was performed to demonstrate the number of myofibroblasts in HBFs and IMR-90 cells. ^**^p < 0.01 compared with NT group; ^&&^*p* < 0.01 compared with shNC+TGF-β1 group; ^@^*p* < 0.05 compared with vector+TGF-β1 group. (B) The protein levels of collagen I and α-SMA in HBFs and IMR-90 cells were evaluated via western blot analysis. ^**^p < 0.01 compared with NT group; ^&&^*p* < 0.01 compared with shNC+TGF-β1 group; ^@@^*p* < 0.01 compared with vector+TGF-β1 group. (C) IF staining was perfoemed to measure the protein content and distribution of α-SMA in HBFs and IMR-90 cells

Furthermore, we intended to explore whether SMOC2 regulated FMT of lung fibroblasts. As shown in Fig. 3A, the results from flow cytometry analysis demonstrated that compared with normal HBFs and MR-90 cells, the number of myofibroblasts (HBFs: from 16.35 to 67.71%; MR-90: from 19.44 to 70.92%) was elevated by TGF-β1 treatment. SMOC2 deficiency offset the TGF-β1-induced increase in the number of myofibroblasts (HBFs: 44.87%; MR-90: 42.34%), whereas SMOC2 elevation further increased (HBFs: 77.72%; MR-90: 84.69%) the number of myofibroblasts in TGF-β1-treated HBFs and MR-90 cells (Fig. 3A). Moreover, we measured the expression of FMT related proteins (collagen I and α-SMA) in HBFs and MR-90 cells via western blot analysis. The data elucidated that the protein levels of collagen I (6- or 12-fold change) and α-SMA (6.9- or 6.5-fold change) were increased in HBFs and MR-90 cells resulted from the treatment of TGF-β1. SMOC2 deficiency led to 0.3- or 0.1-fold reduction of collagen I expression and 0.4- or 0.3-fold reduction of α-SMA expression, while SMOC2 overexpression enhanced collagen I (1.3- or 1.2-fold change) and α-SMA (1.3- or 1.2-fold change) expression in the TGF-β1-treated HBFs and MR-90 cells (Fig. 3B). In addition, IF staining vividly showed that α-SMA was mainly located in cytoplasm, and α-SMA expression was increased in the HBFs and MR-90 cells following TGF-β1 treatment. Up-regulation of α-SMA in the TGF-β1-treated HBFs and MR-90 cells was repressed by SMOC2 depletion, and strengthened by SMOC2 enhancement (Fig. 3C). To sum up, SMOC2 regulated FMT of lung fibroblasts.

### SMOC2 controlled the activation of AKT and ERK

**Fig. 4:**
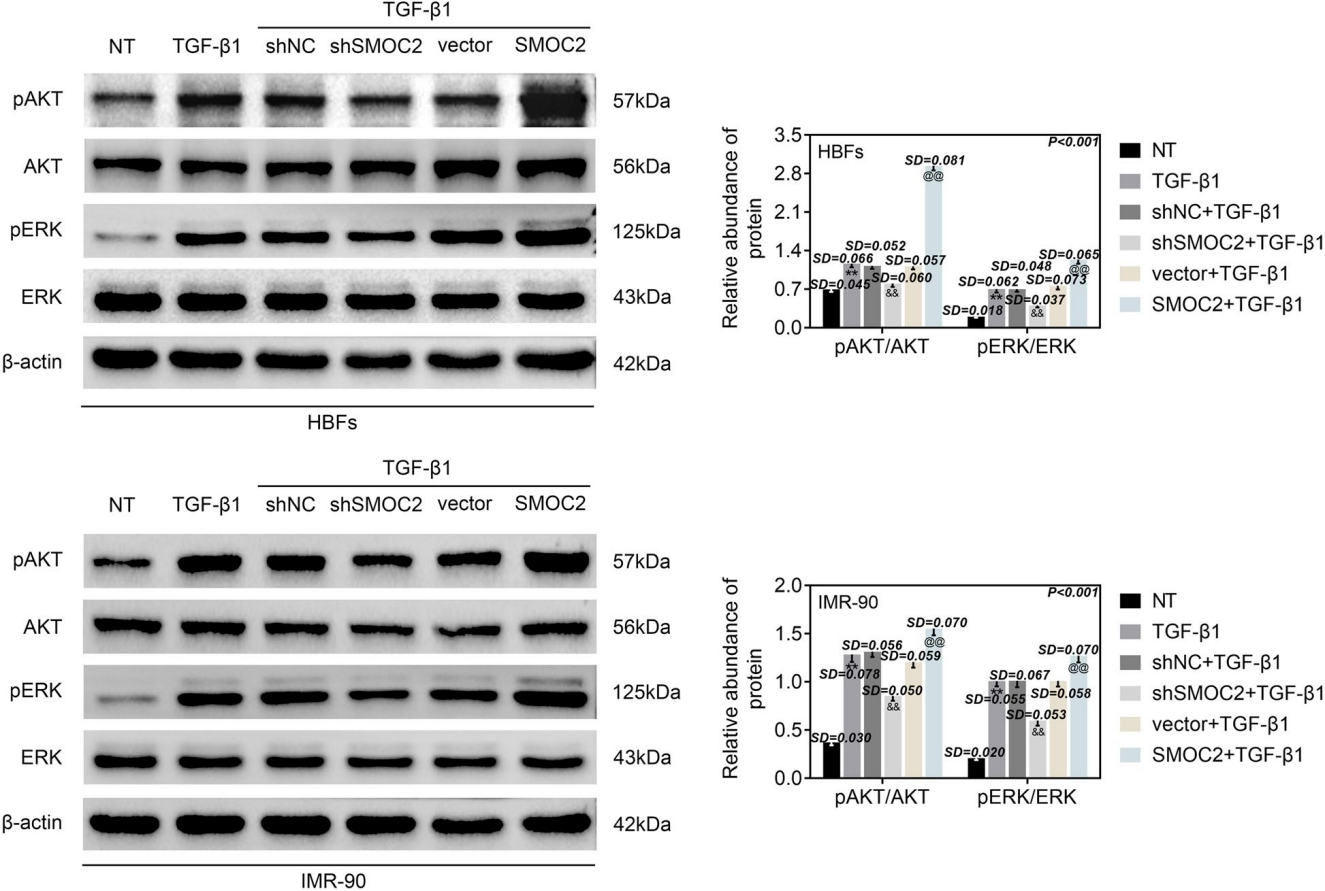
SMOC2 controlled the activation of AKT and ERK. HBFs and IMR-90 cells were transfected with shNC, shSMOC2, vector or SMOC2, and then treated with TGF-β1. Western blot analysis was implemented to evaluate the protein levels of p-AKT and p-ERK in HBFs and IMR-90 cells. ^**^p < 0.01 compared with NT group; ^&&^*p* < 0.01 compared with shNC+TGF-β1 group; ^@@^*p* < 0.01 compared with vector+TGF-β1 group

We were curious about whether SMOC2 could affect AKT and ERK pathways in asthma, and thus we examined the expression of AKT and ERK pathway-related proteins through western blot analysis. The total protein levels of AKT and ERK had no change in the HBFs and MR-90 cells. TGF-β1 treatment elevated the protein levels of p-AKT and p-ERK in HBFs and MR-90 cells, which was abrogated by transfection of shSMOC2. These proteins were up-regulated by overexpression of SMOC2 in TGF-β1-treated HBFs and MR-90 cells (Fig. 4). In conclusion, SMOC2 controlled the activation of AKT and ERK pathways.

### Inhibition of AKT and ERK pathway reversed the promoting effect of SMOC2 overexpression on FMT

**Fig. 5:**
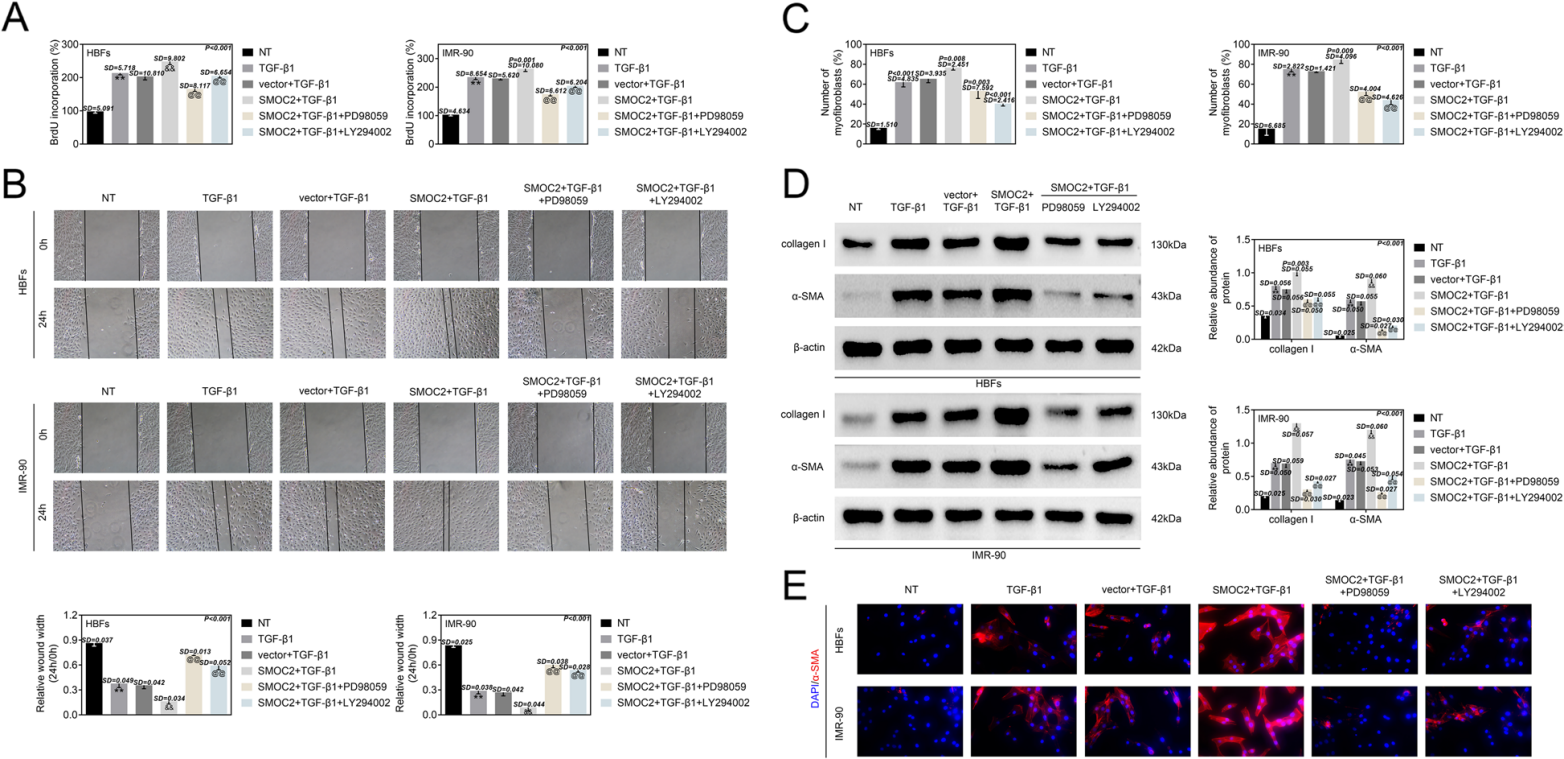
Inhibition of AKT and ERK pathway reversed the promoting effect of SMOC2 overexpression on FMT. HBFs and IMR-90 cells were transfected with vector or SMOC2, and then treated with TGF-β1 or combined with PD98059 or LY294002. BrdU (A) and wound healing (B) assays were performed to assess cell proliferation and migration in HBFs and IMR-90 cells. ^**^p < 0.01 compared with NT group; ^&&^*p* < 0.01 compared with vector+TGF-β1 group; ^@@^*p* < 0.01 compared with SMOC2 + TGF-β1 group. (C) The number of myofibroblasts in HBFs and IMR-90 cells was detected by flow cytometry analysis. ^**^p < 0.01 compared with NT group; ^&&^*p* < 0.01 compared with vector+TGF-β1 group; ^@@^*p* < 0.01 compared with SMOC2 + TGF-β1 group. (D) The protein levels of collagen I and α-SMA in HBFs and IMR-90 cells were measured by western blot analysis. ^**^p < 0.01 compared with NT group; ^&&^*p* < 0.01 compared with vector+TGF-β1 group; ^@@^*p* < 0.01 compared with SMOC2 + TGF-β1 group. (E) IF staining was performed to examine the protein content and distribution of α-SMA in HBFs and IMR-90 cells

Subsequently, the inhibitor of AKT (LY294002, 10 μM) and the inhibitor of ERK (PD98059, 60 μM) were employed to investigate whether SMOC2 regulated proliferation and FMT via activation of AKT and ERK pathways. BrdU assay indicated that SMOC2 upregulation increased cell proliferation ability in TGF-β1-stimulated HBFs and MR-90 cells, which was counteracted by LY294002 or PD98059 treatment (Fig. 5A-B). Moreover, SMOC2 overexpression elevated migration ability (wound width: HBFs: from 0.83 to 0.16; MR-90: from 0.83 to 0.13) in TGF-β1-stimulated HBFs and MR-90 cells. The increase migration ability conferred by SMOC2 overexpression was offset by LY294002 (wound width: HBFs: 0.73; MR-90: 0.64) or PD98059 (wound width: HBFs: 0.65; MR-90: 0.57) treatment. In addition, the number of myofibroblasts was increased in the TGF-β1-treated HBFs and MR-90 cells following transfection of SMOC2 overexpression vector. LY294002 or PD98059 treatment abrogated the boost number of myofibroblasts due to SMOC2 overexpression in TGF-β1-treated HBFs and MR-90 cells (Fig. 5C). Besides, WB analysis and IF staining results all showed that the expression of collagen I (2.3- or 3.4-fold change) and α-SMA (10- or 5-fold change) were increased in HBFs and MR-90 cells following TGF-β1 treatment. SMOC2 overexpression led to 1.3- or 1.8-fold up-regulation of collagen I and 1.6- or 1.7-fold increase of α-SMA expression in the TGF-β1-treated HBFs and MR-90 cells. The expression of collagen I (0.6- or 0.2-fold change) and α-SMA (0.2-fold change) were down-regulated by PD98059 treatment in the TGF-β1-treated HBFs and MR-90 cells. LY294002 treatment caused 0.6- or 0.3-fold reduction of collagen I expression and 0.2- or 0.4-fold decrease of α-SMA expression in the HBFs and MR-90 cells (Fig. 5D-E). Furthermore, Fig. S1 revealed that SMOC2 overexpression enhanced the expression of p-AKT and p-ERK in TGF-β1-treated HBFs and MR-90 cells. The up-regulation of p-AKT and p-ERK by SMOC2 overexpression was reversed by the treatment of LY294002 or PD98059. Overall, inhibition of AKT and ERK pathway reversed the promoting effect of SMOC2 overexpression on FMT.

## Discussion

Asthma is one of the prevalence chronic inflammatory diseases that can lead to coughing, chest tightness or breathlessness [19]. It has become a major chronic disease that seriously threatening public health in the world, the pathogenesis of asthma is still elusive. Previous study has confirmed that the increasing number of proteins have been validated to exert role in asthma, for example, FGF1 accelerates the proliferation and migration of airway smooth muscle cells in asthma [20]. RORγt, as a target of microRNA-17, can modulate the Treg/Th17 cell balance in asthma patients [21]. Nonetheless, the exact role of SMOC2 in asthma is still largely unknown. In the current study, we found the expression of SMOC2 was upregulated by TGF-β1 induction in lung fibroblasts. Moreover, SMOC2 promoted the proliferation and migration of lung fibroblasts. In summary, SMOC2 exerted a pivotal role in asthma.

TGF-β1 has been reported to play a significant part in the pathogenesis of FMT in pulmonary fibrosis. For example, TGF-β mediates the differentiation of airway FTM and contributes to the progression of asthma [22]. The proliferation of MRC-5 cells and overexpression of α-SMA and COL1α1 are triggered by TGF-β1, indicating FTM is induced by TGF-β1 in pulmonary fibrosis [23]. The process of FMT is induced by the activation of TGF-β1 and is associated with the remodeling in asthma [24]. Myofibroblasts, have high proliferation and migration ability, are a kind of transitional cells between the fibroblasts and the smooth muscle cells with an indicator α-SMA [25]. In this work, we found the FMT of lung fibroblasts was regulated by SMOC2. High expression of SMOC2 promoted the FMT in lung fibroblasts.

AKT and ERK pathways are typical pathways that have been verified to be key players in multiple diseases. For example, ERK and AKT signaling pathways are suppressed by the inhibition of PAK1, which decreases the non-small cell lung cancer cell proliferation and invasion [26]. ERK or AKT silencing inhibits intimal hyperplasia in balloon injury rat model through Cx37 upregulation and Cx43 downregulation [27]. AKT/ERK signaling pathways are involved in pulmonary angiogenesis by attenuating the release of NO or NOS in experimental hepatopulmonary syndrome mice [28]. In this study, we explored the downstream pathways of SMOC2 and found the activation of AKT and ERK pathways were affected by SMOC2. Thus, we suspected that SMOC2 might regulate the proliferation, migration and FMT of lung fibroblasts via AKT and ERK pathways. The data indicated that the levels of p-AKT and p-ERK were regulated by SMOC2 in lung fibroblasts. Rescue assays revealed that the treatment of the inhibitor of AKT (LY294002) and the inhibitor of ERK (PD98059) reversed the promoting effect of SMOC2 overexpression on proliferation, migration and FMT in lung fibroblasts. Taken together, SMOC2 influenced asthma progression via the activation of AKT and ERK pathways.

This work demonstrated that SMOC2 modulated TGF-β1-induced proliferation, migration and FMT in lung fibroblasts and may promote asthma, which attributed to activation of AKT and ERK pathways. Thus, this work might offer a promising target molecule for asthma treatment. Nevertheless, there are shortcomings in this study. This article only explored the mechanism of action of SMOC2 in asthma through in vitro experiments. We will construct a mouse model of asthma to further explore the functional role of SMOC2 in asthma in vivo.

## Conclusion

In a word, our study found that SMOC2 participated in TGF-β1-induced proliferation, migration and FMT in lung fibroblasts, which might aggravate the progression of asthma. The results highlight the role of SMOC2 in asthma, which might offer a promising target molecule for asthma treatment.

## Methods

### Cell culture

Human bronchial fibroblasts (HBFs) and human embryonic lung fibroblasts (IMR-90) were obtained from Procell (Wuhan, China). HBFs cells were incubated in DMEM containing 10% fetal bovine serum (FBS; Gibco, Carlsbad, CA, USA) in a humidified atmosphere with 5% CO_2_ at 37 °C, while IMR-90 cells were cultured in MEM supplemented with Earle Salts medium (PAA Laboratories GmbH, Austria), 10% FBS (Gibco), 10 μM sodium pyruvate (10 μM; PAA), gentamycin (125 μg/ml, Krka d.d., Novo Mesto, Slovenia) and non-essential amino acids (10 μM; PAA). Furthermore, different concentrations (1, 5 and 10 ng/mL) of human recombinant TGF-β1 (BD Biosciences, Franklin Lakes, NJ, USA) were added 24 h after cells were planted. The inhibitor of AKT (LY294002, 10 μM) and the inhibitor of ERK (PD98059, 60 μM) were added to the medium 1 h prior to TGF-β1.

### Cell transfection

Short hairpin RNA targeting SMOC2 (shSMOC2) and SMOC2 overexpression plasmid pcDNA3.1/SMOC2 (SMOC2) were bought from GenePharma (Shanghai, China). ShSMOC2 targeting SMOC2 was transfected into HBFs and IMR-90 cells for downregulation of SMOC2, and shNC was employed as a negative control. The pcDNA3.1/SMOC2 was transfected in to HBFs and IMR-90 cells to overexpress SMOC2 with empty vector as a negative control. Cells at the concentration of 1 × 10^5^/100 μL were transfected with 0.8 μg plasmids/shRNAs using 2 μL Lipofectamine 2000 (Invitrogen). After 48 h, cells were collected for further use.

### Real-time quantitative polymerase chain reaction (RT-qPCR)

Total RNA was isolated from HBFs and IMR-90 cells via Trizol reagent (Invitrogen). Complementary DNA (cDNA) was generated with a reverse transcription kit (Roche, Switzerland). Real-time PCR was conducted via FastStart Universal SYBR Green Master (ROX) (Roche) on ABI PRISM 7500 Real-time PCR System (Applied Biosystems, US) in triplicate. The 2^−ΔΔCT^ method was applied for caculating the relative levels of SMOC2 with GAPDH used for normalization. The primers of SMOC2 were presented as follows:

- SMOC2
- F 5′ -AGGAAAAACAGTGATGCCGC-3′
- R 5′ -AACTGCCTTCGGGGTATGAG-3′
- GAPDH
- F 5′ -CTCCTCCTGTTCGACAGTCAGC-3′
- R 5′ -CCCAATACGACCAAATCCGTT-3′

### Immunofluorescence (IF) staining

Myofibroblasts were determined based on immunodetection of α-SMA according to previous studies [29]. Briefly, HBFs and IMR-90 cells were seeded on glass coverslips and fixed in 3.7% paraformaldehyde followed by permeabilising in 0.1% Triton X-100, blocking with 1% BSA. Next, a mouse monoclonal antibody against human α-SMA (clone 1A4, Sigma-Aldrich) and Alexa Fluor 488 goat anti-mouse IgG (clone A11001, Sigma-Aldrich) were cultured with the cells. Thereafter, the specimens mounting in polyvinyl alcohol (Mowiol; Sigma-Aldrich) were visualized via a Leica DMIRE2 microscope. The fluorescence intensity of α-SMA was analyzed using Image J software (National Institute of Health, Bethesda, MD, USA).

### Western blot analysis

RIPA lysis buffer (Beyotime, China) was used for isolating proteins from HBFs and IMR-90 cells. The concentration of protiens was quantified using BCA kit (Beyotime Biotechnology). Afterwards, equivalent amount of proteins (25 μg) was subjected to SDS-PAGE followed by transferring on the polyvinylidene difluoride membranes. Blocked by 5% skim milk at room temperature for 1 h, the membranes were incubated with special primary antibodies including SMOC2 (1:1000, ab56088; Abcam), collagen I (1:1000, ab34710; Abcam), α-SMA (1:500, ab7817; Abcam), AKT (1:10000, ab179463; Abcam), p-AKT (1:500, ab38449; Abcam), ERK (1:1000, ab109282, Abcam) and p-ERK (1:1000, ab214036; Abcam) at 4 °C overnight. Furthermore, it was incubated for 2 h with horseradish peroxidase (HRP) conjugated secondary antibodies at room temperature. At last, the protein bands were assessed by the ECL kit (Thermo Scientific, Waltham, MA, USA). The intensity of the bands was analyzed using Image J software. The expression of proteins was normalized for β-actin.

### Wound-healing assay

HBFs and IMR-90 cells were grown on 12-well plates. After indicated treatment, a linear wound was made by a 200-μL pipette tip. Then, 50 μg/ml mitomycin C was supplemented for 1 h. Afterwards, phosphate-buffered saline (PBS) was applied to wash the cells for three times followed by filling with medium including 1% FBS. The wound was detected via the phase-contrast microscopy using Image J software at 0 h and 24 h. Relative wound width = scratch width at 24 h/scratch width at 0 h.

### Bromodeoxyuridine assay

The proliferation of HBFs and IMR-90 cells was assessed by a cell proliferation enzyme-linked immunosorbent assay BrdU kit (catalogue no. 11647229001; Roche, Mannheim, Germany). After seeding on 96-well plates, HBFs and IMR-90 cells were allowed incubated for 24 h. Finally, a microplate spectrophotometer (Victor 1420 Multilabel Counter, PerkinElmer) was used for measuring the 490 nm absorbance and the absorbance values of BrdU-labeled cellular DNA content indicated the proliferation of HBFs and IMR-90 cells.

### Flow cytometry analysis

The number of myofibroblasts was assessed by flow cytometry analysis. After transfection, HBFs and IMR90 cells were grown into DMEM with additional 10% FBS for one night followed by incubating in serum-decreased medium contianing TGF-β1 or not. After trypsinizing, fibroblasts were subjected to immunostaining and then fixed by paraformaldehyde and permeabilised by Triton X-100. Anti-α-SMA (1:500, ab7817; Abcam) was utilized for evluating the α-SMA expression with mouse-IgG2a isotype served as a control. Cell suspensions were further rinsed by PBS and stained with secondary FITC-conjugated goat anti-mouse IgG (Sigma-Aldrich) for 45 mins. Flow cytometry BD FACS CantoTM II was applied for data collection and FlowJo V10 software (Tree Star, Inc., Ashland, OR, USA) was utilized for data analysis.

### Statistical analysis

Data analysis was conducted via SPSS 21.0 software (IBM, Armonk, NY, USA). The representative data were shown as mean ± standard deviation. The student’s *t* test was utilized to comparison differences between two groups. One-way analysis of variance (ANOVA) followed by LSD post hoc analysis was applied for calculating the differences among mutiple groups. All the experiments were carried out for three times. The value of *p* < 0.05 was considered statistically significant.

## Supplementary Information

**Figure S1 **The changes of AKT or ERK pathway after treating with PD98059 or LY294002. HBFs and IMR-90 cells were transfected with vector or SMOC2, and then treated with TGF-β1 or combined with PD98059 or LY294002. Western blot analysis was used to measure the protein levels of p-AKT and p-ERK in HBFs and IMR-90 cells. ^*^p < 0.05, ^**^p < 0.01 compared with NT group; ^&&^*p* < 0.01 compared with vector+TGF-β1 group; ^@^*p* < 0.05 compared with SMOC2 + TGF-β1 group.

## Data Availability

All data generated or analyzed during this study are included in this published article.

## Abbreviation

TGF-β: transforming growth factor-β
FMT: fibroblast to myofibroblast transformation
SMOC: Secreted modular calcium-binding protein 2
α-SMA: α-smooth muscle actin
ECM: extracellular matrix
SPARC: secreted protein acidic and rich in cysteine
