# Supplementary figures and images for: TGF-β1/SMOC2/AKT and ERK axis regulates proliferation, migration, and fibroblast to myofibroblast transformation in lung fibroblast, contributing with the asthma progression
Source: Hereditas. 2021 Dec 8;158:47. doi: 10.1186/s41065-021-00213-w (PMC8653533; doi:10.1186/s41065-021-00213-w)

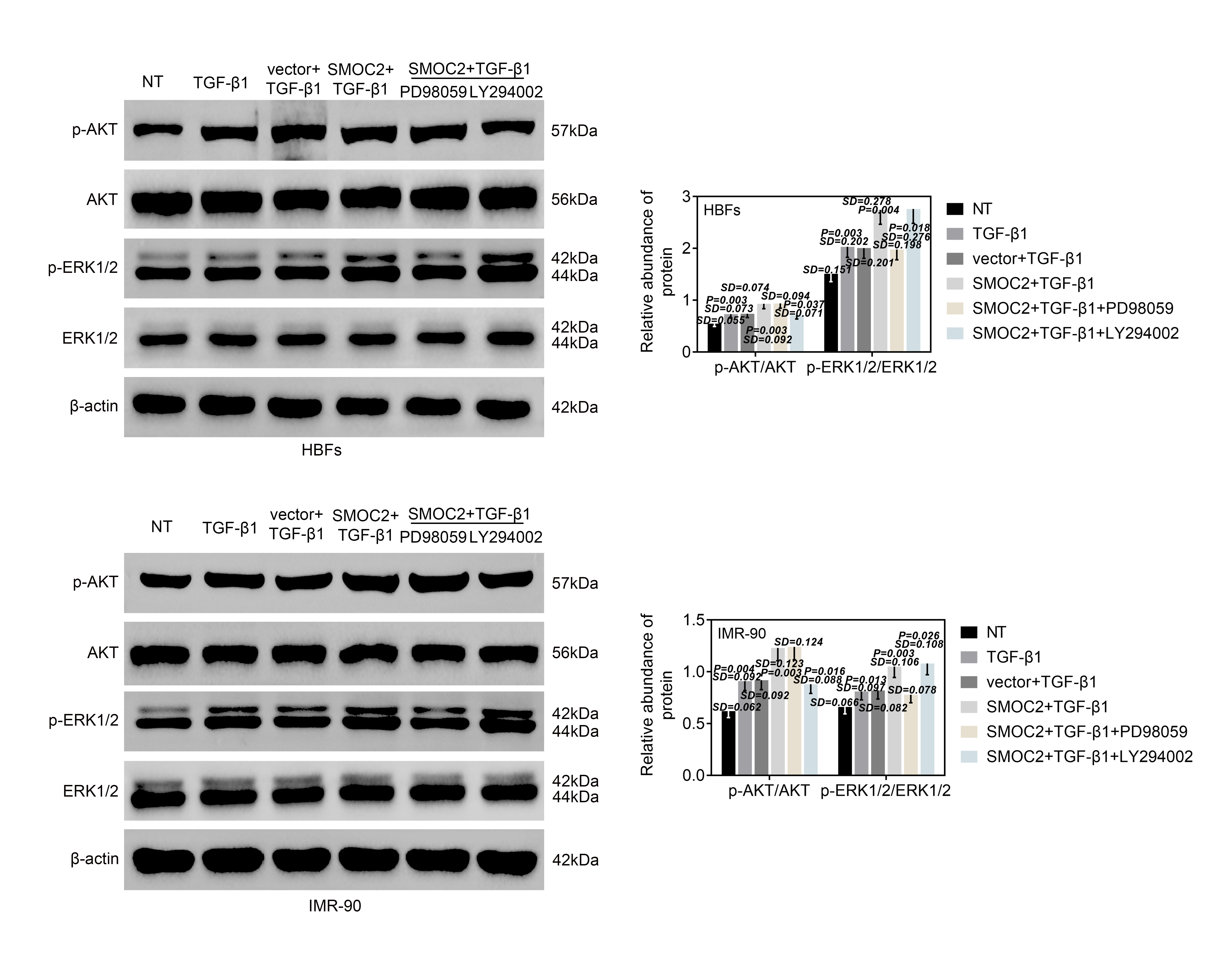

Supplement: Supplementary file 1 — Figure S1 The changes of AKT or ERK pathway after treating with PD98059 or LY294002. HBFs and IMR-90 cells were transfected with vector or SMOC2, and then treated with TGF-β1 or combined with PD98059 or LY294002. Western blot analysis was used to measure the protein levels of p-AKT and p-ERK in HBFs and IMR-90 cells. *p < 0.05, **p < 0.01 compared with NT group; &&p < 0.01 compared with vector+TGF-β1 group; @p < 0.05 compared with SMOC2 + TGF-β1 group. [file 41065_2021_213_MOESM1_ESM.jpg]
